# The utilization of clinical decision support tools to identify neonatal hypothermia and its associated risk factors: A prospective observational study

**DOI:** 10.1371/journal.pgph.0000982

**Published:** 2023-02-09

**Authors:** Mary Kakuru Muhindo, Joshua Bress, Jean Armas, Elon Danziger, Andrew Wu, Ryan C. L. Brewster

**Affiliations:** 1 Infectious Diseases Research Collaboration, Kampala, Uganda; 2 Global Strategies, Albany, California, United States of America; 3 Division of Critical Care Medicine, Boston Children’s Hospital, Boston, Massachusetts, United States of America; 4 Department of Pediatrics, Boston Children’s Hospital, Boston, Massachusetts, United States of America; 5 Department of Pediatrics, Boston Medical Center, Boston, Massachusetts, United States of America; PLOS: Public Library of Science, UNITED STATES

## Abstract

Hypothermia (axillary temperature less than 36.5°) is a major source of neonatal morbidity and mortality, with a disproportionate burden of disease in low- and middle-income countries. Despite the importance of thermoregulation on newborn outcomes, the global epidemiologic landscape of neonatal hypothermia is poorly characterized. Clinical decision support (CDS) software provides point-of-care recommendations to guide clinical management and may support data capture in settings with limited informatics infrastructure. Towards this end, we conducted a prospective observational study of the NoviGuide, a novel CDS platform for newborn care, at four health facilities in Uganda between September 2022 to May 2021. Data were extracted from clinical information (e.g. axillary temperature, birth weight, gestational age) entered into the NoviGuide by nurses and midwives on newborns within 24 hours of delivery. Descriptive statistics and multivariable logistic regression were used to evaluate neonatal temperature profiles and the association between hypothermia and clinical features. Among 1,027 completed assessments, 30.5% of entries had neonatal hypothermia with significant variation across study sites. On multivariable logistic regression analysis, we found that hypothermia was independently associated with pre-term birth (Adjusted Odd’s Ratio [aOR] 2.62, 95% Confidence interval [CI] 1.38–4.98), sepsis/concern for sepsis (aOR 2.73, 95% CI 2.90–3.94), and hypoglycemia/concern for hypoglycemia (aOR 1.78, 95% CI 1.17–2.72). Altogether, neonatal hypothermia was commonly entered into the NoviGuide and associated clinical characteristics aligned with previous studies based on conventional data collection instruments. Our results should be contextualized within unique technical and operational features of CDS tools, including a bias towards acutely ill patients and limited quality control. Nonetheless, this study demonstrates that a CDS used voluntarily by clinicians has the potential to fill key data gaps and drive quality improvement towards reducing neonatal hypothermia in low resource settings.

## Introduction

Neonatal hypothermia, defined as an axillary temperature of less than 36.5°C, is a major contributor to preventable morbidity and mortality. The global burden of hypothermia is concentrated in low- and middle-income countries (LMICs), with prevalence estimates as high as 85% among hospitalized newborns [1]. Although causality is difficult to ascertain, it is a major comorbidity associated with leading causes of death, namely sepsis, prematurity, and asphyxia [2, 3]. Detecting, treating and preventing neonatal hypothermia aligns with the Sustainable Development Goals (SDG) to reduce neonatal (SDG Target 3.2) mortality [4].

A central challenge to addressing neonatal hypothermia is that it is most prevalent in settings with poor record-keeping systems; temperature information is not routinely documented, and the use of electronic medical records are far from universal [5, 6]. Studies that have evaluated neonatal hypothermia in LMICs have frequently relied on independent data collection instruments. These protocols often require more frequent assessments than routine vital sign measurements. Furthermore, evaluations are typically limited to admission temperatures, which is neither representative of nor pragmatic in most birth facilities globally [7–10]. An added complexity is that normothermia is a common performance metric, raising concerns about incentivizing the underreporting of abnormal temperature measurements.

The primary function of clinical decision support software (CDS) is to provide clinical recommendations based on patient-specific parameters and evidence-based guidelines [11]. Some CDS, particularly those that operate as standalone platforms, have begun to integrate data collection functionalities. CDS used voluntarily by clinicians has the potential to enhance our understanding of neonatal hypothermia by capturing temperature profiles in settings with limited data infrastructures. Conversely, where medical records do exist, CDS data can be leveraged to interrogate documentation accuracy and quality.

To date, the characteristics of data derived from a CDS in LMIC, particularly as it concerns neonatal hypothermia, have not been well-defined. In this prospective observational study, we analyze the 1,027 CDS entries submitted by a cohort of midwives and nurses at four rural hospitals in eastern Uganda using the NoviGuide 2.2 application, a novel CDS for newborn care [12]. We aimed to describe the profile of temperatures entered into the NoviGuide and evaluate clinical features associated with neonatal hypothermia.

## Materials and methods

### NoviGuide 2.2 Neonatal Application

The NoviGuide 2.2 Neonatal Application (hereafter referred to as “NoviGuide”) is a neonatal CDS generated with the NoviGuide software, a comprehensive platform for the design, deployment, and monitoring of CDS pathways [12]. Key pathways guide users through detailed, step-by-step evaluations of newborn clinical characteristics, including gestational age, respiratory distress, vital signs, oxygen saturation levels, glucose levels, neonatal infection, human immunodeficiency virus exposure, intravenous fluids, and feeding. Protocols are aligned with national standards using the NoviGuide Algorithm Architect and feature complex branching logic that opens and closes lines of questioning in response to danger signs.

The NoviGuide is not a medical record and does not contain any patient identifiable information. The study team downloaded the NoviGuide onto Amazon Fire HD 8 tablets that were issued to study sites for incorporation into clinical practice. Usage data–including clinical information entered into the Initial Assessment pathway—was stored locally on tablets and synchronized to a cloud-enabled database when connected to a WiFi signal.

### Study design, setting, and participant recruitment

This was a prospective, observational study conducted at four government-owned health facilities located in the Tororo district of eastern Uganda. The hospitals include Tororo General Hospital (TGH), a 200-bed district-level hospital, and three smaller sub-county facilities in Mulanda, Nagongera, and Mukujju sub-counties. Collectively, these facilities serve a population of 597,500 people, the majority of whom reside in rural areas. At the time of data collection, TGH was the only site with a dedicated newborn nursery. All facilities were stocked with basic equipment for neonatal care, including axillary digital thermometers. There are approximately 400 monthly newborn deliveries at TGH, and between 70–100 at the Mulanda, Nagongera, and Mukujju health centers.

We screened and enrolled midwives and nurses at each of the study sites between September 2020 and May 2021. Of note, there was a delay in implementation at the subcounty facilities due to national travel restrictions related to the COVID-19 pandemic. Each participant provided written informed consent and underwent a 4-hour training by NoviGuide trainers on the appropriate use of the platform. NoviGuide Trainers and the study team remained on site for the first two weeks of implementation to aid with workflow integration and troubleshoot user concerns. This study was nested in a larger investigation to evaluate the performance of the NoviGuide under real-world conditions.

### Data collection

Data were collected and abstracted from the most commonly used assessment type, which was the Initial Assessment of newborns under 24 hours of age. We did not apply any eligibility criteria regarding newborn or maternal characteristics. Accordingly, all entries into the NoviGuide application in which the study participant voluntarily completed the Initial Assessment pathway to aid in newborn care were included in the analysis. The user interface for entering the temperature is a slider, which can be adjusted either by dragging the “thumb” within a predefined track, or by pressing a plus or minus symbol to increment or decrement the temperature.

### Exposure variables and outcomes

The primary outcome was neonatal hypothermia within 24 hours of delivery, as documented on the Initial Assessment pathway. Nurses and midwives recorded the axillary temperature using a lithium battery-operated digital thermometer. We described grades of hypothermia in accordance with World Health Organization (WHO) classifications: normal (36.5–37.4°C), mild (36–36.4°C), moderate (32–35.9°C), and severe (<32°C) [13].

We selected covariates for hypothermia *a priori* from NoviGuide usage data, based on known risk factors and associations. These included: gestational age, birth weight, respiratory distress, sepsis or concern for sepsis, and laboratory proven hypoglycemia or symptoms consistent with hypoglycemia (S1 Table). WHO guidelines were used to define low birth weight (<2.5 kg) and pre-term gestational age <37 weeks) [14]. Of note, a key functionality of the NoviGuide is that it provides immediate feedback on user entries, highlighting potentially discordant or unlikely data as it is entered.

### Statistical analysis

We used descriptive statistics with Chi-square tests for neonatal temperature profiles across clinical characteristics. Categorical data were summarized as proportions. Our analysis was limited to only complete assessments (i.e. the user reached the end of the assessment). We evaluated associations of clinical characteristics with neonatal hypothermia with multivariable logistic regression analysis. Statistical significance was defined at a p-value of less than 0.05. A Bonferroni corrected was applied to account for multiple comparisons. All statistical analyses were performed in R, version 3.5.2.

### Ethics statement

The parent study protocol was approved by Makerere University School of Biomedical Sciences (SBS-717), Uganda National Council for Science and Technology (HS 2739) and Western Institutional Review Board (20–29924). The nurses and midwives provided written informed consent before participation in the study-related activities.

## Results

### Study site and newborn characteristics

**Fig 1:**
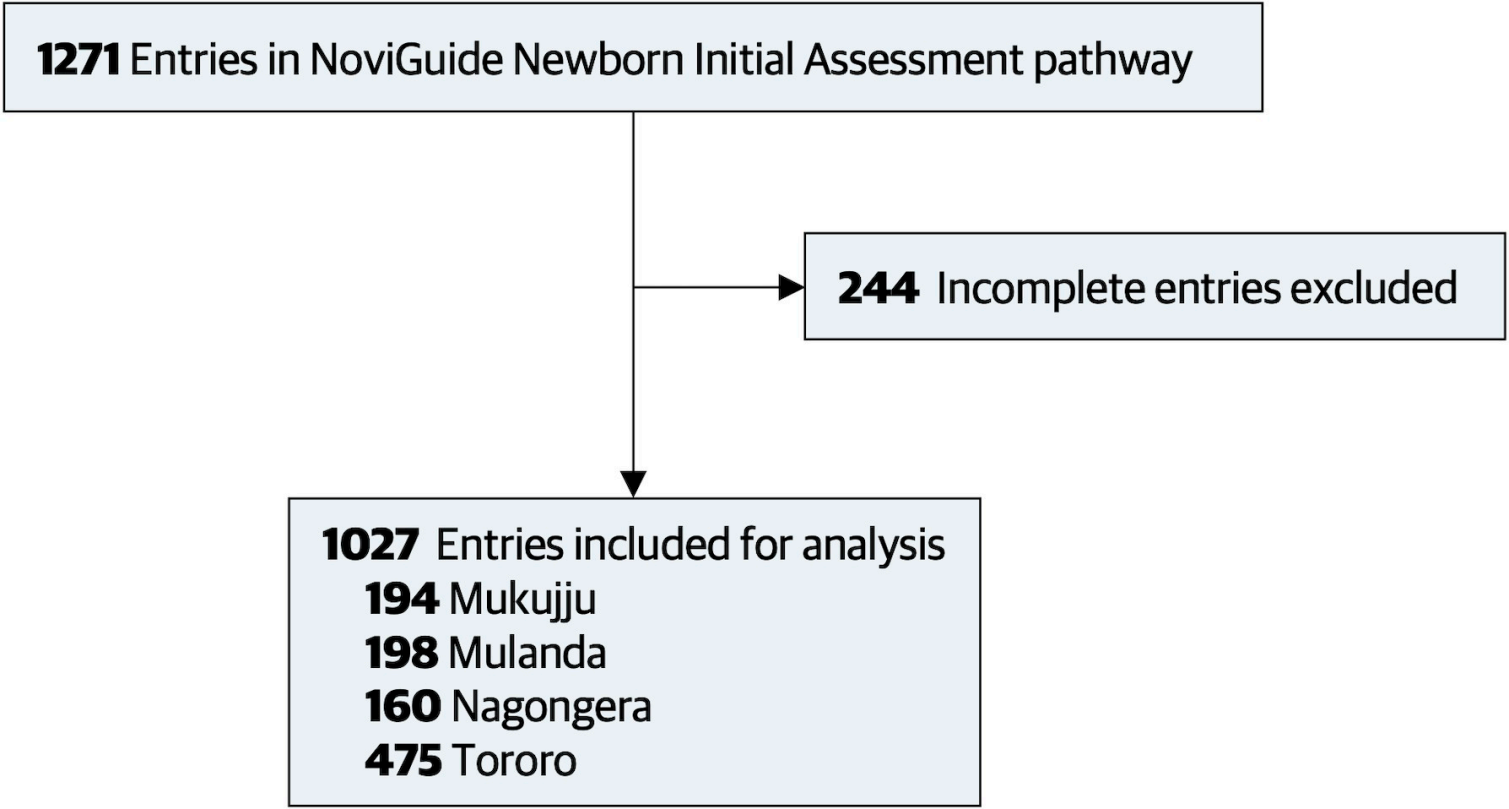
Study flow diagram for NoviGuide usage data.

Fifty-one midwives and nurses completed a total of 1,027 initial newborn assessments between September 2020 and November 2021 (Fig 1) (S2 Table). Most infants entered into the NoviGuide Initial Assessment pathway were born at term (88.6%, n = 910) and had a normal birth weight (82.6%, n = 848). The majority of assessments were performed at Tororo General Hospital (46.3%, n = 475). A total of 244 newborn assessments were started, but not completed, and were therefore excluded from the analysis. Differences in clinical characteristics between complete and incomplete entries are presented in S3 Table. Tororo General Hospital was the first site to adopt the NoviGuide platform (September 2020), followed by Mulanda (March 2021), Nagongera (April 2021), and Mukujju (May 2021). Once introduced, the NoviGuide was available throughout the study period at each study site without interruptions to usage.

### Hypothermia

The median temperature was 36.8°C (Interquartile range 36.3–37°C) with a mode of 37.0°C (S1 Fig). Overall, neonatal hypothermia was recorded in 30.5% (n = 313) of CDS entries (Table 1). Among assessments with neonatal hypothermia, 79.5% and 20.5% had mild (36–36.5°C) and moderate (32–35.9°C) hypothermia, respectively. No CDS entries had severe hypothermia (<32°C).

**Table 1:** Clinical characteristics of study population, by temperature.

	Hypothermic	Normothermic	Febrile	Overall
	N = 313	N = 668	N = 46	N = 1027
**Gestational Age**				
Pre-term (<37 weeks)	77 (24.6%)	34 (5.1%)	3 (6.5%)	114 (11.1%)
Term (≧37 weeks)	233 (74.4%)	634 (94.9%)	43 (93.5%)	910 (88.6%)
Unknown	3 (1.0%)	0 (0%)	0 (0%)	3 (0.3%)
**Birth Weight**				
Low (<2.5 kg)	101 (32.3%)	75 (11.2%)	3 (6.5%)	179 (17.4%)
Normal (≧2.5 kg)	212 (67.7%)	593 (88.8%)	43 (93.5%)	848 (82.6%)
**Respiratory Distress**				
Yes	131 (41.9%)	108 (16.2%)	18 (39.1%)	257 (25.0%)
No	182 (58.1%)	560 (83.8%)	28 (60.9%)	770 (75.0%)
**Sepsis/Concern for Sepsis**				
Yes	241 (77.0%)	267 (40.0%)	45 (97.8%)	553 (53.8%)
No	72 (23.0%)	401 (60.0%)	1 (2.2%)	474 (46.2%)
**Hypoglycemia/Concern for Hypoglycemia**				
Yes	114 (36.4%)	88 (13.2%)	15 (32.6%)	217 (21.1%)
No	199 (63.6%)	580 (86.8%)	31 (67.4%)	810 (78.9%)
**Study Site**				
Mukujju	7 (2.2%)	178 (26.6%)	9 (19.6%)	194 (18.9%)
Mulanda	8 (2.6%)	183 (27.4%)	7 (15.2%)	198 (19.3%)
Nagongera	55 (17.6%)	103 (15.4%)	2 (4.3%)	160 (15.6%)
Tororo	243 (77.6%)	204 (30.5%)	28 (60.9%)	475 (46.3%)

a. Based on clinical evidence, including tachypnea, cyanosis, retractions, oxygen desaturations.

b. Clinical suspicion and presence of risk factors (maternal fever, prolonged rupture of membranes, foul smelling amniotic fluid) warranting initiation of antibiotics.

c. Numerical blood glucose concentration or clinical suspicion.

A higher proportion of preterm patients (<37 weeks gestational age) (24.6% vs. 5.1%) and a lower proportion of term (> = 37 weeks gestational age) patients (74.4% vs 94.9%) were observed among hypothermic patients relative to normothermic patients, respectively (p<0.001) (S2 Table). Similarly, more low birth weight patients (<2.5 kg) (32.3% vs. 11.2%) and fewer normal birth weight (> = 2.5 kg) patients (67.7% vs 88.8%) were observed with neonatal hypothermia than those with normothermia (p<0.001). Patient assessments with respiratory distress (41.9% vs. 16.2%, p<0.001), sepsis/concern for sepsis (77% vs 40%, p<0.001) and hypoglycemia/concern for hypoglycemia (36.4% vs 13.2%, p<0.001) were also noted in greater proportions among patients with neonatal hypothermia than normothermia. There were significant differences in neonatal hypothermia burden across study sites (p<0.001).

On multivariable logistic regression analysis, pre-term gestational age (Adjusted odds ratio [aOR]: 2.62, 95% CI: 1.38–4.98), sepsis/concern for sepsis (aOR: 2.73, 95% CI: 2.90–3.94), and hypoglycemia/concern for hypoglycemia (aOR: 1.78, 95% CI: 1.17–2.72) were among the clinical features associated with hypothermia (Table 2).

**Table 2:** Multivariable logistic regression of clinical characteristics associated with neonatal hypothermia^a^.

	Crude Odds Ratio (95% CI)	Adjusted Odds Ratio (95% CI)
Gestational Age		
Pre-Term (<37 weeks)	**6.16 (4.01–9.48)**	**2.62 (1.38–4.98)**
Term ((≧37 weeks)	Reference	Reference
Birth Weight		
Low (<2.5 kg)	**3.77 (2.69–5.28)**	1.39 (0.82–2.35)
Normal ((≧2.5 kg)	Reference	Reference
Respiratory Distress ^b^		
No	Reference	Reference
Yes	**3.73 (2.75–5.06)**	1.34 (0.89–2.03)
Sepsis/Concern for Sepsis ^c^		
No	Reference	Reference
Yes	**5.03 (3.70–6.82)**	**2.73 (2.90–3.94)**
Hypoglycemia/Concern for Hypoglycemia ^d^		
No	Reference	Reference
Yes	**3.77 (2.73–5.21)**	**1.78 (1.17–2.72)**

a. Statistically significant values are bolded

b. Based on clinical evidence, including tachypnea, cyanosis, retractions, oxygen desaturations

c. Clinical suspicion and presence of risk factors (maternal fever, prolonged rupture of membranes, foul smelling amniotic fluid) warranting initiation of antibiotics

d. Numerical blood glucose concentration or clinical suspicion.

## Discussion

In this prospective, observational analysis of a novel CDS platform in eastern Uganda between September 2020 and November 2021, we found that nurses and midwives frequently entered hypothermic temperatures (30.5%) in a digital pathway for initial newborn assessment. Estimates of neonatal hypothermia extracted from CDS usage data differed across study sites. Additionally, clinical features associated with neonatal hypothermia reflect known risk factors, including prematurity, and symptomatic or laboratory proven hypoglycemia and acute infection. The Noviguide, and CDS more generally, may thus function as a valid and robust tool for data capture and analysis.

There is significant heterogeneity among previous reports of neonatal hypothermia; rates have been found to vary widely in African countries and across diverse geographies, climates, and facilities [9, 15–18]. The lack of comprehensive temperature data globally obscures the epidemiologic picture of neonatal hypothermia and limits the advancement of evidence-based thermoregulatory care in LMIC. Our findings suggest that the NoviGuide has the potential to bridge key data gaps where traditional data collection instruments may not exist [19]. This study builds upon a previous report, which found that the NoviGuide is adaptable, well-received, and significantly improves provider confidence in newborn care [12].

The downstream applications of CDS-derived data extend to both individual facilities and across health systems. Internal review of CDS data could inform indicators for quality improvement initiatives. NoviGuide implementation more broadly could enhance cross-site comparisons, enabling high-risk areas to be more easily targeted. The range in temperature distributions in our study locations, for example, illustrates the importance of high data resolution. More than half of infants were found to be hypothermic at Tororo, compared to less than 5% at Mulanda and Mukujju. The mechanisms underlying site-to-site variation likely encompass both clinic-level factors–such as acuity level, catchment area, and case volumes–and individual-level factors–such as provider usage patterns and experience. As a larger district hospital, Tororo may see a comparatively greater number of home births and medically complex cases, leading to a higher prevalence of hypothermia. Further research is needed to understand how to leverage these insights to support data-driven policy and resource allocation efforts.

Importantly, data captured through CDS usage should be interpreted within unique technical and operational considerations [20]. There are functional differences between CDS software and medical record data. Whereas medical records document clinical care, CDS software serves primarily as an aid in point-of-care decision-making and when used voluntarily, cannot necessarily provide meaningful prevalence or outcome estimates. In our study, CDS usage was dictated by users’ perceived benefit of the technology rather than an administrative mandate. This is reflected in the patient and temperature profiles. Sepsis/concern for sepsis and respiratory distress were more common than what would be expected for a general newborn population [21, 22]. Accordingly, there may be a preferential bias of the NoviGuide towards acutely ill newborns, leading to an overestimation of hypothermia in our cohort.

We also observed a significant number of 37°C temperatures, the default entry in the NoviGuide. Providers may have lacked functioning thermometers or elected not to enter a real temperature. Several quality control steps have been incorporated into the NoviGuide to account for these and other potential biases and discrepancies. Internal logic checks ensure data entries are physiologically consistent and feasible [23]. Users are alerted at the point of care to address incongruencies, such as unlikely weight-for-age combinations. Duplicate entries, numerical outliers, and missing data can be further handled with retrospective aggregate data analysis.

The NoviGuide is specifically designed to improve adherence to clinical guidelines in the settings least able to produce high quality medical records. As a result, research based on CDS usage data is somewhat constrained beyond descriptive studies. We were unable to ascertain the relationship between neonatal hypothermia and mortality, for example, without linkage to individual patient outcomes. This challenge is not easily solved by interoperability alone [24]. Embedding CDS platforms into an electronic medical record, while technically feasible, will not provide the sought-after clarity if documentation is incomplete or of poor quality. Indeed, temperature data is often missing from the medical record in LMICs [25]. Alternatively, introducing novel data collection instruments to measure clinical impact alongside the NoviGuide may distort CDS data if the technology creates a separate clerical burden [26]. As information systems become more accessible globally, integration between CDS, electronic medical records, and other digital platforms should be prioritized to maximize data collection and analysis at a local and public health scale.

## Conclusion

Ugandan nurses and midwives using the NoviGuide frequently documented neonatal hypothermia and usage data closely mirrored known clinical risk factors. These results should be contextualized within intrinsic limitations of data captured through CDS. Neonatal hypothermia is a global problem, but is most severe in settings that also lack high quality medical records and specialized neonatal nurses. By prompting clinicians to take temperatures, alerting clinicians to the presence of hypothermia, guiding corrective action and then gathering data about temperature recordings, CDS may be a useful adjunct to efforts to improve thermoregulation and, by extension, newborn survival.

## Supporting information

S1 Checklist STROBE statement. (DOCX)

S1 Table Definitions of clinical characteristics collected from the NoviGuide. (DOCX)

S2 Table Clinical characteristics of study population, by temperature. (DOCX)

S3 Table Clinical characteristics of study population, by assessment completion status. (DOCX)

S1 Fig Distribution of newborn temperatures entered into the NoviGude, by study site. (TIF)

S1 Data Dataset of midwife and nurse entries into the NoviGuide Initial Assessment pathway. (ZIP)
