# Supplementary material for: The utilization of clinical decision support tools to identify neonatal hypothermia and its associated risk factors: A prospective observational study
Source: PLOS Glob Public Health. 2023 Feb 9;3(2):e0000982. doi: 10.1371/journal.pgph.0000982 (PMC10022021; doi:10.1371/journal.pgph.0000982)
Supplement: S1 Table — (DOCX) [file pgph.0000982.s002.docx]

| **S1 Table.** Definitions of clinical characteristics entered into the NoviGuide | | |
| --- | --- | --- |
| **Clinical Characteristic** | **Description** | **Logic checks** |
| Gestational age | The user first selects from a dropdown menu that includes preterm, term, post-term or unknown. If preterm is selected, the user is then asked to specify an estimated gestational age in weeks. If unknown is selected, the user is prompted to estimate if the newborn is less than 37 weeks and, if so, is treated as preterm above. | The weights and gestational ages are compared at the moment of entry. Users see alert messages for weight-for-age comparisons that are above or below 2 standard deviations and 4 standard deviations using Fenton growth charts. Second, for preterm entries, where the user must designate an age in weeks, the user sees a table that includes the corresponding last menstrual period date for each gestational age. |
| Birthweight | The User enters the weight in format X.XX kg. A number must be entered to proceed with the assessment. | See above regarding weight-for-age logic. Additionally, there are range limits of 0.5 kg - 5.99 kg. |
| Respiratory distress | The User is asked a yes/no question: “Is the baby having difficulty breathing?” | If the user answers ‘no’, but then enters a respiratory rate >60, an alert message is generated to confirm the absence of respiratory distress. Similar logic prompts users who respond ‘no’ to the difficulty breathing question, but then enter in an oxygen saturation <90%. |
| Sepsis or concern for sepsis | We defined sepsis or concern for sepsis as the intention to give intravenous antibiotics as indicated by progression through the CDS to weight and age specific guidance on antibiotic dosing. | In babies who are identified by the user as sick appearing or in respiratory distress or who have risk factors for sepsis, users will see a message informing them of their guidelines for giving antibiotics in those instances. Users can then progress to receiving guidance on dosing or foregoing antibiotics. Users can also indicate they do not have antibiotics. For term babies with no risk factors, Users see a message with information consistent with their guidelines, that well term babies with no risk factors do not routinely need antibiotics. Users can still choose to see antibiotic guidance. For babies who are preterm, but well-appearing, the user is informed that prematurity is itself a risk factor and to strongly consider antibiotics below a gestational age of 34 weeks. Users can then choose to progress to guidance on antibiotic dosing. |
| Hypoglycemia or concern for hypoglycemia | Defined as a glucose level below 45 (if user has a glucometer) or the presence of symptoms of hypoglycemia. | NoviGuide’s branching logic is configured to site-based equipment. Therefore, sites that have indicated they lack glucometers are never prompted to enter a glucose level and instead progress to a symptom-based assessment. |
