# Supplementary material for: The utilization of clinical decision support tools to identify neonatal hypothermia and its associated risk factors: A prospective observational study
Source: PLOS Glob Public Health. 2023 Feb 9;3(2):e0000982. doi: 10.1371/journal.pgph.0000982 (PMC10022021; doi:10.1371/journal.pgph.0000982)
Supplement: S3 Table — (DOCX) [file pgph.0000982.s004.docx]

| **S3 Table.** Clinical characteristics of study population, by assessment completion status | | | |
| --- | --- | --- | --- |
|  | **Incomplete** | **Complete** | **P-value^a^** |
|  | N=235 | N=1027 |  |
| **Temperature** |  |  |  |
| Hypothermic (<36.5°C) | 83 (35.3%) | 313 (30.5%) | **0.002** |
| Normothermic (36.5-37.4 °C) | 103 (43.8%) | 668 (65.0%) |  |
| Hyperthermic (>37.4 °C) | 13 (5.5%) | 46 (4.5%) |  |
| Missing | 36 (15.3%) | 0 (0%) |  |
| **Gestational Age** |  |  |  |
| Pre-term (<37 weeks) | 61 (26.0%) | 114 (11.1%) | **<0.001** |
| Term (≧37 weeks) | 174 (74.0%) | 910 (88.6%) |  |
| Missing | 0 (0%) | 3 (0.3%) |  |
| **Birth Weight** |  |  |  |
| Low (<2.5 kg) | 67 (28.5%) | 179 (17.4%) | **<0.001** |
| Normal (≧2.5 kg) | 168 (71.5%) | 848 (82.6%) |  |
| **Respiratory Distress^b^** |  |  |  |
| Yes | 86 (36.6%) | 257 (25.0%) | **<0.001** |
| No | 149 (63.4%) | 770 (75.0%) |  |
| **Sepsis/Concern for Sepsis^c^** |  |  |  |
| Yes | 76 (32.3%) | 553 (53.8%) | **<0.001** |
| No | 159 (67.7%) | 474 (46.2%) |  |
| **Hypoglycemia/Concern for Hypoglycemia^d^** | | |  |
| Yes | 38 (16.2%) | 217 (21.1%) | 0.351 |
| No | 115 (48.9%) | 810 (78.9%) |  |
| Missing | 82 (34.9%) | 0 (0%) |  |
| **Study Site** |  |  |  |
| Mukujju | 43 (18.3%) | 194 (18.9%) | **<0.001** |
| Mulanda | 13 (5.5%) | 198 (19.3%) |  |
| Nagongera | 32 (13.6%) | 160 (15.6%) |  |
| Tororo | 147 (62.6%) | 475 (46.3%) |  |
| 1. Two-sided chi-square test of association between entry completion status and clinical characteristics. Significant values are bolded. 2. Based on clinical evidence, including tachypnea, cyanosis, retractions, oxygen desaturations 3. Clinical suspicion and presence of risk factors (maternal fever, prolonged rupture of membranes, foul smelling amniotic fluid) warranting initiation of antibiotics 4. Numerical blood glucose concentration or clinical suspicion | | | |
