# Supplementary figures and images for: The utilization of clinical decision support tools to identify neonatal hypothermia and its associated risk factors: A prospective observational study
Source: PLOS Glob Public Health. 2023 Feb 9;3(2):e0000982. doi: 10.1371/journal.pgph.0000982 (PMC10022021; doi:10.1371/journal.pgph.0000982)

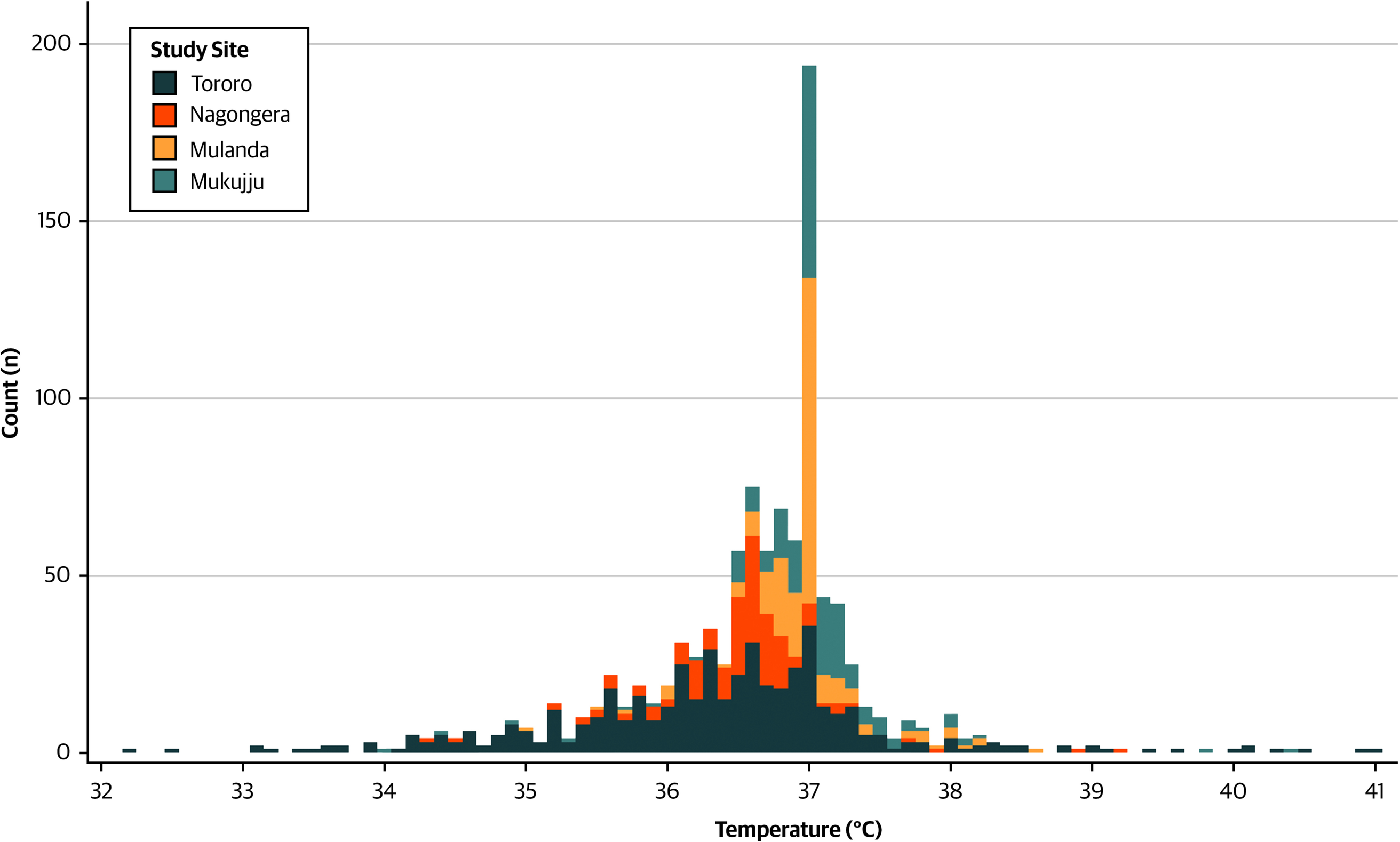

Supplement: S1 Fig — (TIF) [file pgph.0000982.s005.tif]
